# Signal Propagation via Open-Loop Intrathalamic Architectures: A Computational Model

**DOI:** 10.1523/ENEURO.0441-19.2020

**Published:** 2020-02-18

**Authors:** Jeffrey W. Brown, Aynaz Taheri, Robert V. Kenyon, Tanya Y. Berger-Wolf, Daniel A. Llano

**Affiliations:** 1 College of Medicine, University of Illinois at Urbana-Champaign, Urbana, IL 61801; 2 Department of Computer Science, University of Illinois at Chicago, Chicago, IL 60607; 3 Beckman Institute for Advanced Science and Technology, University of Illinois at Urbana-Champaign, Urbana, IL 61801; 4 Neuroscience Program, University of Illinois at Urbana-Champaign, Urbana, IL 61801; 5 Department of Molecular and Integrative Physiology, University of Illinois at Urbana-Champaign, Urbana, IL 61801

**Keywords:** computational model, cortical signaling, intrathalamic signaling, open-loop, propagation, thalamic reticular nucleus

## Abstract

Propagation of signals across the cerebral cortex is a core component of many cognitive processes and is generally thought to be mediated by direct intracortical connectivity. The thalamus, by contrast, is considered to be devoid of internal connections and organized as a collection of parallel inputs to the cortex. Here, we provide evidence that “open-loop” intrathalamic pathways involving the thalamic reticular nucleus (TRN) can support propagation of oscillatory activity across the cortex. Recent studies support the existence of open-loop thalamo-reticulo-thalamic (TC-TRN-TC) synaptic motifs in addition to traditional closed-loop architectures. We hypothesized that open-loop structural modules, when connected in series, might underlie thalamic and, therefore cortical, signal propagation. Using a supercomputing platform to simulate thousands of permutations of a thalamocortical network based on physiological data collected in mice, rats, ferrets, and cats and in which select synapses were allowed to vary both by class and individually, we evaluated the relative capacities of closed-loop and open-loop TC-TRN-TC synaptic configurations to support both propagation and oscillation. We observed that (1) signal propagation was best supported in networks possessing strong open-loop TC-TRN-TC connectivity; (2) intrareticular synapses were neither primary substrates of propagation nor oscillation; and (3) heterogeneous synaptic networks supported more robust propagation of oscillation than their homogeneous counterparts. These findings suggest that open-loop, heterogeneous intrathalamic architectures might complement direct intracortical connectivity to facilitate cortical signal propagation.

## Significance Statement

Interactions between the dorsal thalamus and thalamic reticular nucleus (TRN) are speculated to contribute to phenomena such as arousal, attention, sleep, and seizures. Despite the importance of the TRN, the synaptic microarchitectures forming the basis for dorsal thalamus-TRN interactions are not fully understood. The computational neural model we present incorporates “open-loop” thalamo-reticular-thalamic (TC-TRN-TC) synaptic motifs, which have been experimentally observed. We elucidate how open-loop motifs possess the capacity to shape the propagative properties of signals intrinsic to the thalamus and evaluate the wave dynamics they support relative to closed-loop TC-TRN-TC pathways and intrareticular synaptic connections. Our model also generates predictions regarding how different spatial distributions of reticulo-thalamic and intrareticular synapses affect these signaling properties.

## Introduction

Propagation of activity across the cerebral cortex is thought to underlie multiple cognitive processes, as well as pathologic processes such as epilepsy and migraine (Leao, 1944; Muller et al., 2014, 2016; Kokkinos et al., 2017). Cortical regions are highly interconnected via direct axonal projections as well as via polysynaptic pathways involving the basal ganglia and thalamus (Parent and Hazrati, 1995; Theyel et al., 2010). Cortical signal propagation is generally thought to be mediated via direct cortical connections (Felleman and Van Essen, 1991; Kötter and Sommer, 2000), but recent evidence suggests that the thalamus serves as a control point to modify cortical activity during cognitive processes such as attentional shifting (Wimmer et al., 2015). An advantage of a thalamic mode of signal propagation is the efficiency by which modulatory influences may control thalamic, and therefore cortical, propagation. The thalamus, however, is generally thought to have limited internal connectivity and therefore limited capacity to serve as a substrate for signal propagation.

A major intermediary allowing for communication between thalamocortical neurons, the thalamic reticular nucleus (TRN), is a sheet of GABAergic neurons that partially envelops the dorsal thalamus (Pinault, 2004). It has been speculated to participate in phenomena ranging from selective attention (Crick, 1984; Guillery et al., 1998; McAlonan et al., 2006) to sleep and arousal (Llinás and Paré, 1991, Steriade et al., 1993; Guillery et al., 1998; McAlonan et al., 2006) and fear responses (Dong et al., 2019), and may play a role in generating absence seizures (von Krosigk et al., 1993; Bal et al., 1995; Destexhe et al., 1996a; Huguenard, 1998; McCormick and Contreras, 2001), symptoms of neurodevelopmental disorders (Wells et al., 2016; Krol et al., 2018), and schizophrenia (Ferrarelli and Tononi, 2011). The TRN projects exclusively to thalamic relay (thalamocortical, or TC) neurons, while receiving reciprocal, glutamatergic thalamoreticular (TC-TRN) connections (Sherman and Guillery, 2001).

**Figure 1.**
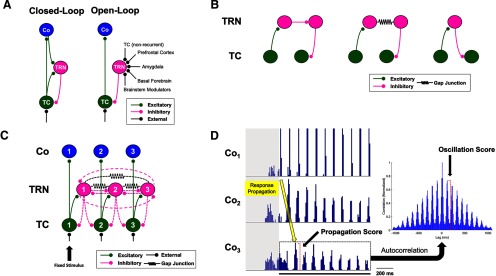
Pathways and properties of thalamocortical signaling. ***A***, Closed-loop versus open-loop TC-TRN-TC configurations. ***B***, Three possible pathways through which a signal might propagate from one TC neuron to another via the TRN. ***C***, Baseline thalamocortical model network. Broken-line synapses were allowed to vary either as a class (homogeneously) or independently of one another (heterogeneously). The black arrow corresponds to the fixed, external stimulus applied to TC_1_. ***D***, Sample cortical spike histograms (detrended) in a network permutation responding to a fixed, sustained stimulus delivered to TC_1_ (black bar beneath the lowest trace). The propagation score assigned to any network permutation was quantified as the amplitude of the initial stimulus-evoked response in the detrended Co_3_ histogram; response propagation across the cortical subnetwork (orange arrow) was consistently linear, and thus the initial response in Co_3_ was observed at a fixed interval relative to the onset of stimulation. Oscillation intrinsic to any network variant was quantified as the amplitude of the first off-center peak in the normalized autocorrelogram (right) of poststimulation activity in the detrended Co_3_ histogram (within the broken black box). The initial 400 ms of activity preceding the fixed stimulus (in gray) is shown here for each histogram but was not included in the calculations of either propagation or oscillation. Note that the bin heights in the Co_1_ histogram shown here were truncated in order to maintain identical vertical scaling across all three cortical histograms.

The structural microarchitecture of bidirectional pathways connecting the dorsal thalamus and TRN has been the subject of ongoing debate. It was originally assumed that thalamo-reticulo-thalamic (TC-TRN-TC) pathways were reciprocal, forming “closed loops” of recurrent inhibition delivered to TC neurons (Fig. 1*A*, left; Hale et al., 1982; Steriade et al., 1993; Warren et al., 1994; Sherman and Guillery, 1996; Pinault, 2004). While closed disynaptic loops have indeed been confirmed, they were only identified in a minority of examined TC-TRN pairs (Shosaku, 1986; Lo and Sherman, 1994; Pinault and Deschênes, 1998; FitzGibbon et al., 2000; Gentet and Ulrich, 2003; Pinault, 2004). Another connectional scheme between the dorsal thalamus and TRN is the so-called “open-loop” TC-TRN-TC pathway, wherein a TC neuron is not reciprocally inhibited by the TRN neuron it excites (Fig. 1*A*, right). Open-loop configurations have been inferred from recordings in rodent thalamic slice preparations (Crabtree et al., 1998; Crabtree and Isaac, 2002; Lam and Sherman, 2005, 2015; Lee et al., 2010) and confirmed in anatomic studies (Pinault and Deschênes, 1998; Kimura et al., 2007; Kimura, 2014). Furthermore, open-loop pathway variants in the form of X-TRN-TC are also known to exist, with X representing indirect sources of modulation to the sensory thalamus via the TRN, including monoaminergic and cholinergic brainstem nuclei, GABAergic nuclei of the basal forebrain, the amygdala, and prefrontal cortex (Morrison and Foote, 1986; Hallanger et al., 1987; Asanuma and Porter, 1990; Bickford et al., 1994; Zikopoulos and Barbas, 2006; Sun et al., 2013; Pita-Almenar et al., 2014; Wimmer et al., 2015).

Based on previous studies of open-loop TC-TRN-TC synaptic organization, we hypothesized that open-loop synaptic modules might underlie intrathalamic and therefore intracortical signal propagation. Accordingly, we systematically examined thousands of permutations of a novel network model comprising thalamic, reticular, and thalamorecipient, layer-4 cortical (Co) neurons to evaluate the efficacy of known thalamic synaptic motifs (open-loop pathways, closed-loop pathways, and chemical and electrical intrareticular synapses), in isolation and in combination, in mediating signal transmission across the thalamus and cortex.

## Materials and Methods

### Network architecture and simulations

We constructed a baseline model network based on Willis et al. (2015) by connecting in series three thalamocortical pathways, each consisting of a TC, TRN, and layer-4 Co neuron (for a three-by-three neuron network); the physiological data used in our model were collected in mice, rats, ferrets, and cats of both sexes. Permutations of the baseline network potentially featured both closed-loop and/or open-loop TC-TRN-TC motifs, with the latter constituting one mode of connectivity between parallel thalamocortical pathways. Intrareticular synapses represented the other connections between pathways, based on the identification of both GABAergic (Ahlsén and Lindström, 1982; Steriade et al., 1990; Cox et al., 1996; Sanchez-Vives et al., 1997; Shu and McCormick, 2002; Deleuze and Huguenard, 2006; Lam et al., 2006) and electrical synapses (Landisman et al., 2002; Fuentealba et al., 2004; Long et al., 2004; Deleuze and Huguenard, 2006; Lam et al., 2006) between TRN neurons. Thus, we included three different polysynaptic configurations between thalamocortical pathways in our network (Fig. 1*B*, from left to right): (1) those with a GABAergic intrareticular synapse (TRN-TRN_GABA_), (2) those with an electrical intrareticular synapse (TRN-TRN_Elec_), and (3) open-loop TC-TRN-TC pathways. Thalamic, reticular, and cortical cell layers were aligned topographically, such that TC_1_ projected to both TRN_1_ and Co_1_ (Jones, 1975; Steriade et al., 1993; Destexhe et al., 1998; Sohal et al., 2000; Sherman and Guillery, 2001). The divergence of thalamic and reticular synapses in the model was constrained to accommodate open-loop TC-TRN-TC architectures, which depend on a lack of recurrent feedback to the downstream TC neuron, in a subset of the simulated network variants: to this end, every TC neuron projected to exactly one TRN neuron, while single TRN neurons could project to either one TC neuron (whether recurrently or laterally), as in the case of entirely closed-loop or open-looped TC-TRN-TC motifs, or two TC neurons (one recurrently and one laterally), if participating in a pathway expressing some intermediate degree of openness (Fig. 1*C*).

To analyze how each variety of interpathway connection contributed to network dynamics, permutations of the baseline network were generated by varying three synaptic properties associated with each of the interpathway synaptic motifs; moreover, these parameters were either varied in a homogeneous or heterogeneous manner. In the case of homogeneously varied synaptic network permutations, the synaptic parameters associated with three interpathway motifs varied uniformly as a class, with all external, TC-TRN, and thalamocortical (TC-Co) synaptic conductances held constant: (1) TRN-TRN_GABA_ synapses ranged in conductance between 0 and 450 nS, (2) TRN-TRN_Elec_ synapses ranged in coupling coefficient between 0 and 0.36, and (3) a TC-TRN-TC “openness” coefficient, defined as the weight distribution of lateral versus recurrent reticulothalamic (TRN-TC) connectivity, varied between 0 (completely closed-loop) and 1.0 (completely open-loop) and with a baseline TRN-TC conductance of 80 nS. Thus, for a network variant possessing an openness coefficient of 0.4 (i.e., exhibiting slightly more closed-loop than open-loop TC-TRN-TC connectivity), the laterally inhibitory TRN-TC synapses in the network, TRN_1_→TC_2_ and TRN_2_→TC_3_, would carry a conductance of 32 nS (0.4 × 80 nS), while the recurrent TRN-TC synapses, TRN_1_→TC_1_, TRN_2_→TC_2_, and TRN_3_→TC_3_, would exhibit a conductance of 48 nS (0.6 × 80 nS). For the heterogeneously varied synaptic network variants, all TRN-TRN and TRN-TC synapses were allowed to vary independently. Domains for each of the synaptic variables were selected to include the range of conductance or coupling strengths reported in physiological measurements and/or used in similar neural models (Destexhe et al., 1996a, 1998; Sohal and Huguenard, 1998; Sohal et al., 2000; Landisman et al., 2002; Long et al., 2004; Traub et al., 2005).

Ongoing afferent synaptic input was delivered to every TC neuron in the model as Poisson-modulated spike trains centered at 40 Hz. An additional 200-Hz pulse train was applied to neuron TC_1_ between *t *=* *0.400 s and *t *=* *1.500 s during every network simulation run. This high-frequency stimulus was modeled on those used to elicit spindle-like waves in a ferret thalamoreticular slice preparation (Bal et al., 1995; Kim et al., 1995). A given network’s output was compiled by assembling spike histograms (10-ms bins) averaging 1000 simulations for every cortical neuron (Fig. 1*D*). We quantified network dynamics as a function of variable TC-TRN-TC and intrareticular synaptic architectures by defining and measuring two properties inherent to stimulus-evoked responses in each network variant: propagation and oscillation, with the latter included in light of the fact that many characterized thalamic waveforms both oscillate and propagate through the thalamus and cortex (Sherman and Guillery, 2001). Network properties were quantified in the most downstream element of the cortical output layer, Co_3_. Propagation across a network was quantified as the amplitude of the initial stimulus-evoked response in the detrended Co_3_ histogram. The degree of oscillation supported by each network permutation was defined as the amplitude of the first off-center peak in the normalized autocorrelogram of poststimulation activity (Fig. 1*D*). Both propagation and oscillation scores are reported as normalized to the maximum scores tabulated for each property. Given the high prevalence of propagating oscillatory waves in the cerebral cortex (for review, see Muller et al., 2018), we furthermore defined a composite “optimization” (*Op*) metric to measure the capacity of networks to simultaneously support and balance between propagation (*Pr*) and oscillation (*Os*):

(1) $$Op=\sqrt{Pr^{2}+Os^{2}}-\left|Pr-Os\right|.$$

### Intrinsic neuronal models

Single-compartment TC, TRN, and cortical model neurons obeyed Hodgkin–Huxley kinetics, with membrane potentials *V* varying according to the first-order differential equation:

(2) $$C\frac{dV}{dt}=-g_{L}\left(V-E_{L}\right)-\underset{i}{\sum }g_{i}\left(V\right)\left(V-E_{i}\right),$$

where *C* is the membrane capacitance, *g_L_* and *E_L_* are the leakage conductance and reversal potential, respectively, and *g_i_(V)* and *E_i_* are the dynamic conductance and reversal potential, respectively, of the *i*th voltage-gated, ligand-gated (chemical synaptic), or electrical synaptic conductance (for electrical synaptic conductances, the effective reversal potential is equal to the presynaptic membrane potential; see Eq. 3a). All three varieties of model neurons expressed both the standard transient sodium (*I_Na_*) and delayed-rectifier potassium (*I_K_*) currents. TC and TRN neurons additionally included a T-type calcium conductance (T-current; *I_T_*) and hyperpolarization-activated cation current (H-current; *I_H_*), following the TC model of Deleuze et al. (2012). Both TRN and layer-4 Co cells expressed a slow, non-inactivating potassium conductance (*I_M_*), following the modeling of Pospischil et al. (2008), which accounts for the spike-frequency adaptation previously reported in physiological recordings from these neurons (Yamada et al., 1989; Willis et al., 2015). A list of intrinsic model cell parameters, including current conductances, reversal potentials, selected gating kinetics, and membrane capacitance, can be found in Table 1.

**Table 1:** Intrinsic model cellular parameters

Intrinsic model cellular parameters			
Parameter	TC cell	TRN cell	Co cell
Leak conductance, *g_L_* (nS)	3.263	3.7928	4.8128
Leak reversal potential, *E_L_* (mV)	–60.03	–57	–60.2354
Transient sodium conductance, *g_Na_* (nS)	1500	3000	3000
Sodium equilibrium potential, *E_Na_* (mV)	50	50	50
Delayed-rectifier potassium conductance, *g_K_* (nS)	520	400	140
M-type potassium conductance, *g_M_* (nS)	-	3.5	1.5
M-type potassium time constant, τ_M_ (ms)	-	200	180
Potassium equilibrium potential, *E_K_* (mV)	–100	–100	–90
T-type calcium conductance, *g_T_* (nS)	45	21	-
Calcium equilibrium potential, *E_T_* (mV)	120	120	120
H-current conductance, *g_H_* (nS)	0.608	0.0192	-
H-current reversal potential, *E_H_* (mV)	–33	–33	-
Membrane capacitance, *C_m_* (pF)	100.4	75.0	109.3865

### Synaptic models

The kinetics of chemical synapses in our model network conformed to the synaptic depression model of Tsodyks and Markram (1997). This model presupposes a finite quantity of “resources,” akin to synaptic vesicles, capable of being released by the presynaptic neuron; these resources can exist in an active, inactive, or recovered state. A parameter *U_SE_* characterizes the fraction of recovered resources that can be converted to an active state (i.e., for release by the presynaptic neuron) following action potential induction in the presynaptic axon terminal (s). Following resource activation, synapses inactivate according to the time constant τ_inact_; resources become available again for activation after a recovery period described by the time constant τ_recov_. These parameters, along with the neurotransmitters, postsynaptic conductances, and reversal potentials characterizing all of the chemical synapses in our model, are given in Table 2.

**Table 2:** Model synaptic parameters

Model synaptic parameters						
Synapse	Neurotransmitter	Conductance (nS)	τ_recov_ (ms)	τ_inact_ (ms)	Reversal potential (mV)	*U_SE_*
External synapse to TC cell	(Glutamate)	32	125	2.64	0	0.76
TC-to-TRN cell synapse (TC-TRN)	Glutamate	150	500	2.64	0	0.76
TC-to-Co cell synapse (TC-Co)	Glutamate	50	160	11.52	0	0.8113
TRN-to-TC cell synapse (TRN-TC)	GABA_A_	Variable (0–80)	167.29	16.62	–80	0.62
Chemical TRN-to-TRN cell synapse (TRN-TRN_GABA_)	GABA_A_	Variable (0–450)	225	15	–75	0.62

**τ**_recov_, synaptic recovery time constant; **τ**_inact_, synaptic recovery time constant; *U_SE_*, fraction of recovered resources (synaptic vesicles) that can be converted to an active state (Tsodyks and Markram, 1997).

Glutamatergic TC-TRN and TC-Co and baseline GABAergic TRN-TC synaptic parameters matched those of Willis et al. (2015), with the latter synapses allowed to vary in conductance as described above. TRN-TC signaling was mediated exclusively through GABA_A_ receptors, mirroring other thalamic and thalamocortical models in which the slower TRN-TC GABA_B_ conductance was omitted (Traub et al., 2005; Rogala et al., 2013; Pham and Haas, 2018). Although evidence has been presented challenging the existence of GABAergic intrareticular synapses in certain mammalian species and age groups (Pinault et al., 1997; Landisman et al., 2002; Pinault, 2004; Cruikshank et al., 2010; Hou et al., 2016), our model avoided making assumptions regarding their presence, strength, or spatial distribution by allowing the associated synaptic conductances to vary over a range of physiological values, including zero, and in distribution. The reversal potential, conductance, and kinetics of the external synapses projecting to the TC neurons were directly based on retinogeniculate synapses (Chen and Regehr, 2003), although the generic nature of the external inputs in our model allows them to represent not only immediately upstream sensory input but also brainstem modulation (e.g., serotonergic, adrenergic) known to act on thalamic nuclei (Siegel and Sapru, 2015).

Electrical synapses between TRN neurons were based on the Cx36-dependent intrareticular gap junctions first identified by Landisman et al. (2002). For TRN neurons, the sum of electrical synaptic currents (*I_Elec_*) entering any postsynaptic neuron *j* from presynaptic neuron(s) *i* was included in the rightmost term from Equation 2 and calculated as

(3a) $$I_{Elec\left(j\right)}=\underset{i}{\sum }g_{ij}\left(V_{j}-V_{i}\right),$$

where *g_ij_*, the gap junction conductance, was itself calculated as

(3b) $$g_{ij}=D(x)\frac{g_{j}}{1/\mathrm{CC}-1},$$

where *CC* was the electrical coupling coefficient between TRN neurons *i* and *j*, *g_j_* was the membrane conductance of the postsynaptic neuron, and *D(x)* was a scaling factor that depended on the physical distance between the coupled TRN neurons (Dayan and Abbott, 2005; Shimizu and Stopfer, 2013). Although the individual gap junctions comprising TRN-TRN_Elec_ synapses used in the model were not explicitly coded for, differences in TRN-TRN coupling between different electrical synapses and network permutations implicitly reflected differing gap junction densities, following Traub et al. (2005). TRN-TRN_Elec_ synapses were symmetrical (non-rectifying), such that *g_ij_=g_ji_*.

We extrapolated the attenuation of intrareticular synaptic strength as a function of intracellular distance based on mappings of intrinsic connections within the TRN along a horizontal (anteroposterior) plane assembled by Deleuze and Huguenard (2006). Assuming (1) an intracellular distance of 50 μm between adjacent TRN neurons, (2) a distance *x* (in multiples of 50 μm) between non-adjacent neurons, and (3) a Gaussian falloff in synaptic strength (Sohal et al., 2000), the baseline (adjacent-neuron) conductances of TRN-TRN_GABA_ and TRN-TRN_Elec_ synapses were scaled for non-adjacent synapses using the function

(4) $$D\left(x\right)=e^{-\frac{x^{2}}{2\lambda^{2}}},$$

where λ_GABA_ = 531 μm and λ_Elec_ = 130 μm.

Given the small spatial scale of our model, synaptic delays associated with finite axonal conductance times within the TRN and between the TRN and dorsal thalamus were disregarded, mirroring the simplification incorporated into previous thalamic and thalamocortical models simulating synaptic interactions on the order of 100 μm (Golomb et al., 1996; Traub et al., 2005). Although small (∼1 ms) thalamocortical delays were inserted into the network model of Traub et al. (2005), these were likewise omitted on the basis of the cortex functioning solely as an output layer in our model.

### Quantification and statistical analysis

Our model was coded, simulated, and analyzed in MATLAB R2018b (MathWorks) using both a Dell Inspiron 3847 and Hewlett-Packard Z840 running Windows 10 and nodes on the Illinois Campus Cluster (National Center for Supercomputing Applications, University of Illinois at Urbana-Champaign). Simulations, of which there were 1000 for every network permutation, employed 0.1-ms time steps, with temporal integration based on the hybrid analytic-numeral integration method of Moore and Ramon (1974), which optimizes between accurate solutions to Hodgkin–Huxley and synaptic models and computational efficiency. All simulations commenced with a 200-ms equilibration period, during which no external stimulation was delivered to TC neurons; this allowed all network elements to attain steady-state conditions. The number of homogeneously and heterogeneously varied synaptic network variants generated were 770 and 12,681, respectively.

Statistical analysis was performed in both MATLAB and R (R Core Team, 2013), with the *glmnet* package (Friedman et al., 2010) used within the latter platform to perform regression analyses. Multiple linear regression was employed to establish rudimentary relationships between synaptic classes (homogeneously synaptic networks) or individual synapses (heterogeneously synaptic networks) and each of the two studied network properties, even in instances where these relationships deviated from linearity. Second-order polynomial (2°) regression models with interaction terms elucidated how synaptic interactions and nonlinearities affected these network properties. Regressions were optimized using elastic net regularization, with the specific regularization hyperparameter α selected to minimize each regression model’s root-mean-square error (RMSE). To convey the relative influence of different synaptic classes or individual synapses on dynamic network properties, all regression coefficients are reported here as normalized to the coefficient with the largest absolute value; the effects corresponding to normalized regression coefficients (NRCs) with absolute values of <0.05 were disregarded as negligibly influential on network dynamics. Both unpaired Student’s *t* tests and one-way analysis of variance models were used to compare the mean property scores between different sets of networks, with Tukey’s honestly significant difference tests used to ascertain pairwise difference between groups in the latter; standard errors of the mean (SEMs) were used as a measure of variance, and null hypotheses were rejected at probability values (*p* values) below 0.05. Kolmogorov–Smirnov and Levene’s tests were employed to confirm normality and homogeneity of variance, respectively, when using parametric mean-comparison tests; data were transformed as needed to conform to these prerequisites.

### Code accessibility

The code/software described in the paper is freely available online at https://github.com/JeffreyWBrown/Open-loop-TC-TRN-TC. The code is available as Extended Data 1.

10.1523/ENEURO.0441-19.2020.ed1 Extended Data 1 Raw and analyzed data and simulation and analysis code generated during this study. Download Extended Data 1, ZIP file.

## Results

### Propagation and oscillation in homogeneously varied synaptic models

**Figure 2.**
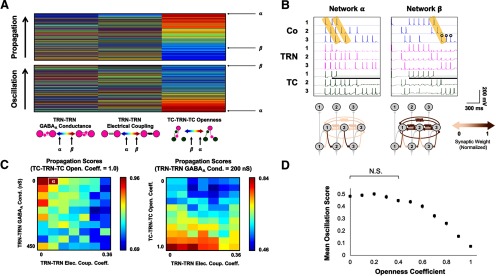
Propagation and oscillation in homogeneously varied synaptic networks (*N* = 770). ***A***, Ordinal heat maps ranking homogeneously varied synaptic network permutations according to the extent of supported signal propagation and oscillation. Every row in a given map depicts a single network permutation, color-coded based on its synaptic makeup according to the three synaptic scales found below the maps (redder colors signify stronger or more open-loop connections). The network property ranks and synaptic makeups of two selected networks, Networks α and β, are indicated. ***B***, Representative simulations and circuit diagrams depicting the normalized synaptic makeups for the two selected networks. The black bar indicates when the fixed stimulus was delivered to TC_1_ in each simulation. Orange highlighting indicates epochs of linear propagation, while circles are placed above spikes occurring during periods of oscillatory activity. ***C***, Heat maps displaying propagation scores in TRN-TRN synaptic parameter space for the 70 fully open-loop networks (openness coefficient = 1.0), with Network α highlighted (left), and propagation as a function of TC-TRN-TC openness and electrical coupling between TRN neurons for the 70 networks possessing 200-nS GABAergic TRN-TRN synapses (right). ***D***, Mean oscillation scores for networks varied nonlinearly as a function of their openness coefficients, with networks possessing openness coefficients of 0 and 0.4 supporting oscillation to equal extents (one-way analysis of variance with Tukey *post hoc* tests, *F*_(10,759)_ = 137.8, *p *<* *0.0001). Individual means were computed by averaging the 70 oscillation scores associated with a given openness coefficients, and error bars indicate SEM; N.S. = not significant.

Stimulus-evoked responses propagated linearly across the length of homogeneous synaptic networks, occurring at average fixed intervals of 93.31 ± 0.35 ms (mean ± SEM; range, 60–110 ms) between adjacent thalamocortical pathways, across all model permutations and with a mean velocity of 0.54 mm/s, assuming a 50-μm separation between adjacent neurons in each network layer. All 770 homogeneous network variants were ranked according to their cortical propagation scores (Fig. 2*A*, top). TC neurons exhibited both tonic firing and bursting activity, with the former mode more frequently observed (Fig. 2*B*).

Multiple linear regression analysis (*R*^2^ = 0.793, RMSE = 0.047, *p *<* *0.0001) demonstrated a strong positive correlation between the TC-TRN-TC openness coefficient and propagation score (NRC = 1.000). By contrast, chemical and electrical TRN-TRN synaptic connectivity tended to modestly diminish propagation (NRC = −0.173 and NRC = −0.136, respectively; Table 3). Further, other excitatory connectivity, such as corticocortical or corticothalamic connectivity, often postulated as being important for cortical signal propagation (Felleman and Van Essen, 1991; Kötter and Sommer, 2000; Theyel et al., 2010), was not necessary. Thus, the homogeneously varied synaptic network permutations that best accommodated signal propagation were generally ones with weak or absent synapses between TRN neurons and strong open-loop TC-TRN-TC connections. For example, Network α, which epitomizes this architecture, exhibited robust signal propagation in response to a fixed stimulus delivered to TC_1_; a representative simulation of this network is shown in Figure 2*B*, left, and its position within the parameter space depicted in the left-sided heat map of Figure 2*C* is labeled. Stimulus-evoked activity in this network tended to propagate efficiently from Co_1_ to Co_3_: near-synchronous propagation cascades were elicited in both the TRN and cortical layers of the model, having been stimulated by propagating activity in upstream TC neurons. Smooth, linear propagation of action potentials across the network depended on the synchronous induction of IPSPs and the ensuing postinhibitory rebound spikes in TC neurons, as mediated by T-type Ca^2+^ channels and driven by inhibition from the TRN, which occurred reliably and at fixed intervals in Network α. Relative to Network α, other network permutations exhibiting stronger intrareticular synapses did not support propagation as efficiently. We surmise that TRN-TRN_GABA_ synaptic connections reduced the incidence of IPSPs in TC neurons required for signal propagation across the network, while electrical coupling between TRN neurons destructively shunted a propagating signal away from the thalamoreticular lattice through which it predominantly traversed the network.

**Table 3.** Normalized linear and 2° regression coefficients for propagation and oscillation in homogeneously varied synaptic networks

Normalized regression coefficients for homogeneously varied synaptic networks				
Synaptic variable	Propagation linear	Propagation 2°	Oscillation linear	Oscillation 2°
TRN-TRN_GABA_	–0.173	–0.670	-	0.060
TRN-TRN_Elec_	–0.136	–0.347	-	-
Open_TC-TRN-TC_	1.000	1.000	–1.000	–0.052
(TRN-TRN_GABA_)^2^	-	0.332	-	-
(TRN-TRN_Elec_)^2^	-	0.164	-	-
(Open_TC-TRN-TC_)^2^	-	0.594	-	–1.000
TRN-TRN_GABA_ × TRN-TRN_Elec_	-	0.262	-	-
TRN-TRN_GABA_ × Open_TC-TRN-TC_	-	–0.152	-	-
TRN-TRN_Elec_ × Open_TC-TRN-TC_	-	–0.365	-	-

The regressions include 1°, 2°, and interaction terms corresponding to TRN-TRN_GABA_, TRN-TRN_Elec_, and TC-TRN-TC openness (Open_TC-TRN-TC_). Terms associated with regression coefficients of absolute values <0.05 are omitted. Linear regression for propagation, *R*^2^ = 0.793, RMSE = 0.047, *p *<* *0.0001; 2° regression for propagation, *R*^2^ = 0.842, RMSE = 0.041, *p *<* *0.0001; linear regression for oscillation, *R*^2^ = 0.526, RMSE = 0.145, *p *<* *0.0001; 2° regression for oscillation, *R*^2^ = 0.630, RMSE = 0.128, *p *<* *0.0001.

A 2° regression model of propagation as a function of all three synaptic class variables (*R*^2^ = 0.842, RMSE = 0.041, *p *<* *0.0001; Table 3) revealed a modestly negative interaction term between TRN-TRN_Elec_ synapses and TC-TRN-TC openness (NRC = −0.365), indicating that in networks where both electrical synapses were strong and TC-TRN-TC openness high, the extent of supported propagation diminished nonlinearly; a smaller negative interaction between TRN-TRN_GABA_ synapses and TC-TRN-TC openness was also observed (NRC = −0.152). Together, these terms suggested that propagation was more significantly affected by connections in the TRN layer within the more open-loop networks. This relationship was evident, for example, in the right-sided heat map of Figure 2*C*, in which propagation scores more markedly decreased with increasing TRN-TRN electrical coupling as TC-TRN-TC openness itself increased.

Oscillatory responses recurred in Co_3_ neurons at a mean frequency of 9.07 ± 0.2 Hz (range, 7.14–12.50 Hz) across all homogeneous model permutations. Propagation and oscillation scores across all 770 homogeneous networks were strongly anticorrelated (Pearson’s *r* = −0.671, *p *<* *0.0001). Accordingly, oscillation was best accommodated in network permutations exhibiting strongly closed-loop connectivity (Fig. 2*A*, bottom); however, the capacity to support oscillation was neither markedly linear nor monotonically decreasing as a function of increasing openness coefficient (Fig. 2*D*). Rather, a one-way analysis of variance with Tukey’s tests revealed that, on average, oscillation scores peaked and remained statistically indistinguishable from one another across the subset of network permutations with openness coefficients between 0 and 0.4, with scores then decreasing in a roughly linear fashion with increasing TC-TRN-TC openness (*F*_(10,759)_ = 137.8, *p *<* *0.0001); this result was consistent with a 2° regression model of oscillation (*R*^2^ = 0.630, RMSE = 0.128, *p *<* *0.0001; Table 3), in which the linear and quadratic terms in TC-TRN-TC openness were associated with NRCs of −1.000 and −0.052, respectively, and the effects of TRN-TRN_GABA_ (NRC = 0.060) and TRN-TRN_Elec_ (NRC regularized to 0) on oscillation were weakly positive and negligible, respectively. Taken with the analysis of propagation, these data suggest that networks with mixed open- and closed-loop connectivity (which is likely close to physiological reality) can support the coexistence of oscillation and propagation (see below, Propagation and oscillation in heterogeneously varied synaptic models).

The predominant mechanism by which oscillation arose in Co_3_ was through postinhibitory rebound in TC_3_, as engendered by the strong recurrent inhibition found in network permutations exhibiting primarily closed-loop TC-TRN-TC connectivity. This mode of oscillation was exemplified by Network β, a strongly closed-loop network variant. In the simulation shown of this network (Fig. 2*B*, right), oscillatory activity was enabled by a single epoch of signal propagation. Notably, neither the presence of strong GABAergic nor electrical intrareticular synapses in Network β exerted much effect on its ability to support oscillation, as predicted by the 2° regression model.

### Propagation and oscillation in heterogeneously varied synaptic models

**Figure 3.**
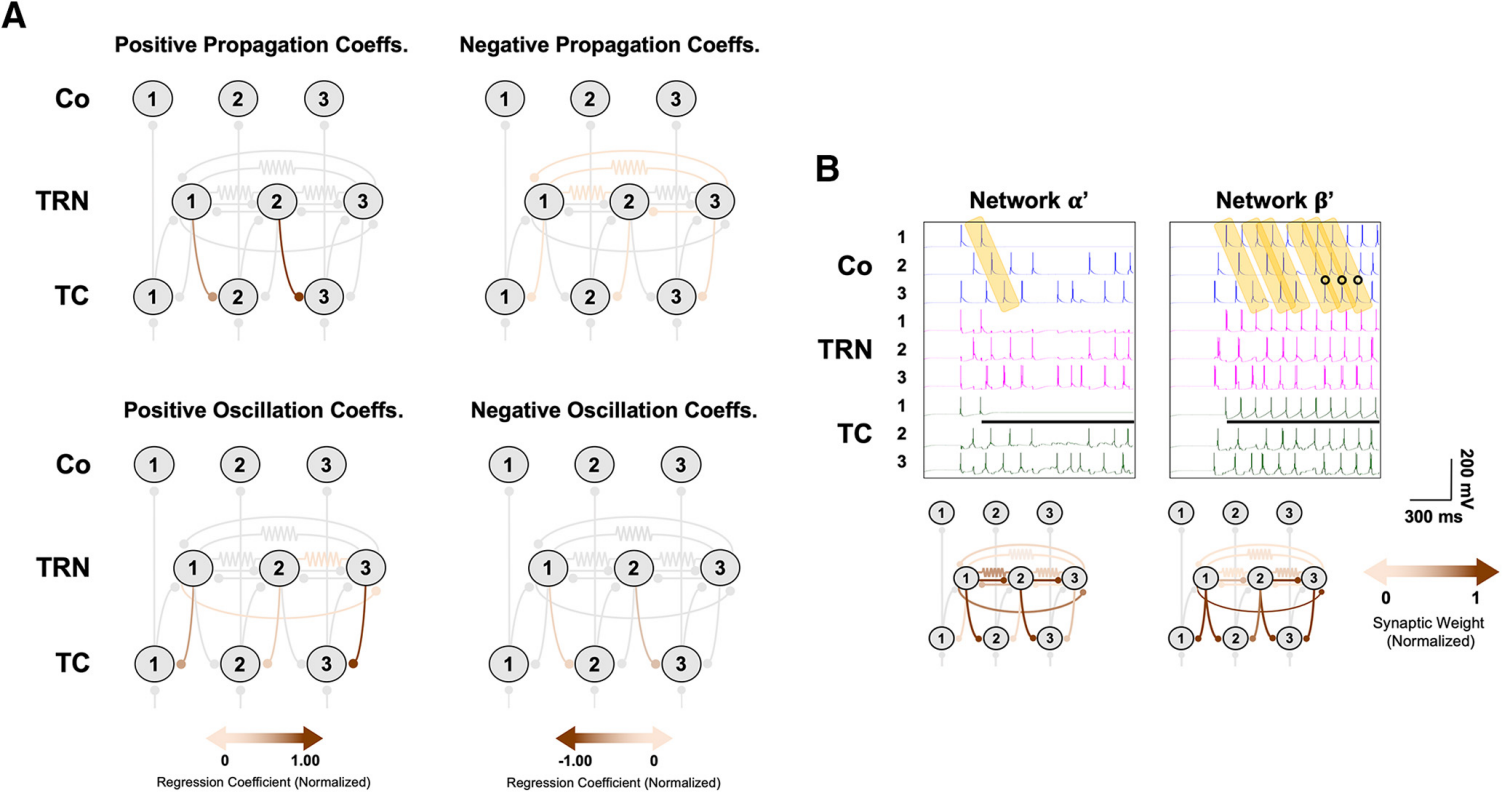
Propagation and oscillation in heterogeneously varied synaptic networks (*N* = 12,681). ***A***, Network regression models illustrating how propagation (top) and oscillation (bottom) varied as a function of individual synaptic weights across simulated heterogeneously synaptic network permutations. Synapses with positive and negative normalized regressions coefficients were correlated positively and negatively with a given property and are depicted separately in the left- and right-sided circuit diagrams, respectively, for clarity. Gray synapses are either non-variable or associated with normalized regression coefficients with absolute values <0.05. See also Table 4. ***B***, Representative simulations for two selected heterogeneous networks, whose normalized synaptic weights are depicted in the circuit diagrams. Networks α‘ and β‘ respectively illustrate propagation and propagation of oscillation across the network.

Recent studies have highlighted heterogeneity in TRN neuronal connectivity, synaptic physiology and chemical identities (Lee et al., 2007; Halassa et al., 2014; Clemente-Perez et al., 2017). We therefore examined the impact of allowing all synaptic connections involving the TRN to be independently varied. We constructed circuit-level schematics of linear regression models for propagation (Fig. 3*A*, top) and oscillation (Fig. 3*A*, bottom) as functions of the 14 synaptic variables in heterogeneous networks.

Propagation in heterogeneously varied synaptic networks increased chiefly as a function of increasing the strength of the more downstream of the two laterally inhibitory TRN-TC synapses, TRN_2_→TC_3_: the corresponding term in a linear regression model of propagation (*R*^2^ = 0.742, RMSE = 0.069, *p *<* *0.0001; Table 4) possessed an NRC of 1.000 (Fig. 3*A*, top). Propagation scores also scaled to a lesser extent with the more upstream laterally inhibitory reticulothalamic synapse, TRN_1_→TC_2_ (NRC = 0.608). The two inhibitory intrareticular synapses originating at the rightmost end of the model network, TRN_3_→TRN_1_ and TRN_3_→TRN_2_, both exerted a small negative effect on propagation (NRC = −0.087 and NRC = −0.084, respectively). Additionally, two TRN-TRN_Elec_ synapses, TRN_1_=TRN_2_ and TRN_1_=TRN_3_ (where the “=” denotes an electrical synapses), marginally decremented propagation in heterogeneous networks, with NRCs of −0.051 and −0.072, respectively. These findings at an individual synaptic level comported with the observation that strong TRN-TRN interactions, whether chemical or electrical, tended to impede signal propagation in homogeneous network variants.

**Table 4:** Normalized linear and 2° regression coefficients for propagation and oscillation in heterogeneously varied synaptic networks

Normalized regression coefficients in heterogeneously varied synaptic networks				
Synaptic variable	Propagation linear	Propagation 2°	Oscillation linear	Oscillation 2°
TRN_1_-TRN_3_	-	-	0.115	-
TRN_3_-TRN_1_	–0.088			
TRN_3_-TRN_2_	–0.084	–0.073	-	-
TRN_1_=TRN_2_	–0.051	–0.091	-	-
TRN_1_=TRN_3_	–0.072	-	-	-
TRN_2_=TRN_3_	-	–0.113	0.117	-
TRN_1_-TC_1_	–0.075	-	0.621	0.077
TRN_1_-TC_2_	0.608	0.571	–0.289	–1.000
TRN_2_-TC_2_	–0.128	–0.196	0.333	0.417
TRN_2_-TC_3_	1.000	1.000	–0.379	–0.892
TRN_3_-TC_3_	–0.207	–0.239	1.000	0.107
(TRN_3_-TRN_2_)^2^	-	0.079	-	-
(TRN_1_-TC_2_)^2^	-	–0.245	-	0.189
(TRN_2_-TC_2_)^2^	-	0.174	-	–0.093
(TRN_2_-TC_3_)^2^	-	–0.472	-	0.278
(TRN_3_-TC_3_)^2^	-	0.187	-	–0.146
TRN_1_-TRN_2_ × TRN_1_-TC_2_	-	0.070	-	-
TRN_1_-TRN_3_ × TRN_3_-TC_3_	-	-	-	0.215
TRN_2_-TRN_1_ × TRN_1_=TRN_2_	-	-	-	0.111
TRN_2_-TRN_1_ × TRN_1_-TC_1_	-	-	-	–0.186
TRN_3_-TRN_1_ × TRN_1_-TC_1_	-	-	-	–0.172
TRN_3_-TRN_1_ × TRN_1_-TC_2_	-	–0.119	-	-
TRN_3_-TRN_1_ × TRN_2_-TC_3_	-	–0.096	-	-
TRN_3_-TRN_2_ × TRN_2_-TC_3_	-	–0.153	-	-
TRN_1_=TRN_2_ × TRN_2_-TC_3_	-	-	-	–0.129
TRN_1_=TRN_3_ × TRN_1_-TC_1_	-	-	-	–0.114
TRN_1_=TRN_3_ × TRN_3_-TC_3_	-	–0.079	-	-
TRN_1_-TC_1_ × TRN_1_-TC_2_	-	-	-	0.634
TRN_1_-TC_1_ × TRN_2_-TC_3_	-	-	-	0.449
TRN_1_-TC_2_ × TRN_2_-TC_2_	-	–0.166	-	0.361
TRN_1_-TC_2_ × TRN_2_-TC_3_	-	0.753	-	–0.274
TRN_1_-TC_2_ × TRN_3_-TC_3_	-	–0.106	-	0.669
TRN_2_-TC_2_ × TRN_2_-TC_3_	-	-	-	0.345
TRN_2_-TC_2_ × TRN_3_-TC_3_	-	-	-	–0.192
TRN_2_-TC_3_ × TRN_3_-TC_3_	-	–0.124	-	0.399

The regressions include 1°, 2°, and interaction terms corresponding to the 14 variable synapses in the networks. Equal signs denote gap junctions. Linear regression for propagation, *R*^2^ = 0.742, RMSE = 0.069, *p *<* *0.0001; 2° regression for propagation, *R*^2^ = 0.857, RMSE = 0.051, *p *<* *0.0001; linear regression for oscillation, *R*^2^ = 0.253, RMSE = 0.131, *p *<* *0.0001; 2° regression for oscillation, *R*^2^ = 0.388, RMSE = 0.118, *p *<* *0.0001.

A 2° regression model (*R*^2^ = 0.857, RMSE = 0.051, *p *<* *0.0001; Table 4) disclosed a large, propagation-enhancing interaction between the two laterally inhibitory synapses (NRC = 0.753), underscoring the same dependence of propagation on strong open-loop TC-TRN-TC connectivity as seen in homogeneously synaptic networks, but additionally demonstrating that propagation scores increased nonlinearly as a function of simultaneously increasing the weights of TRN_1_→TC_2_ and TRN_2_→TC_3_. Interactions between TRN-TRN synapses of either variety and TRN-TC synapses tended diminish propagation, as did those between recurrent and lateral inhibitory TRN-TC synapses. Taken together, the linear and 2° regression models indicated that heterogeneous network permutations with strong laterally inhibitory TRN-TC synapses tended to best support propagation. Consistent response propagation across the length of the network was epitomized by Network α‘, in which TRN_1_→TC_2_ and TRN_2_→TC_3_ were both relatively strong and those synapses impeding propagation comparatively weak (Fig. 3*B*, left).

### Heterogeneously varied synaptic architectures better supported propagation of oscillation

In contrast to the homogeneous models, there was a very small negative correlation between the propagation and oscillation scores of heterogeneous networks (*r* = −0.0296, *p *=* *0.0008), suggesting that propagation and oscillation more easily coexist in heterogeneous than homogeneous models. This supposition was confirmed through a 2° regression analysis (*R*^2^ = 0.388, RMSE = 0.118, *p *<* *0.0001), which suggested that interactions between recurrently and laterally inhibitory TRN-TC synapses (NRCs ranging between 0.345 and 0.669) facilitated the propagation of oscillation, a mechanism typified by Network β‘ (Fig. 3*B*, right). Two intrareticular synapses, TRN_1_-TRN_3_ and TRN_1_=TRN_3_, tended to contribute modestly to oscillation (NRCs of 0.115 and 0.117, respectively, in the linear regression model, *R*^2^ = 0.253, RMSE = 0.131, *p *<* *0.0001; Fig. 3*A*, bottom), while, in their individual capacities, TRN_1_→TC_2_ and TRN_2_→TC_3_ diminished oscillation (NRCs of −1.000 and −0.892, respectively).

**Figure 4.**
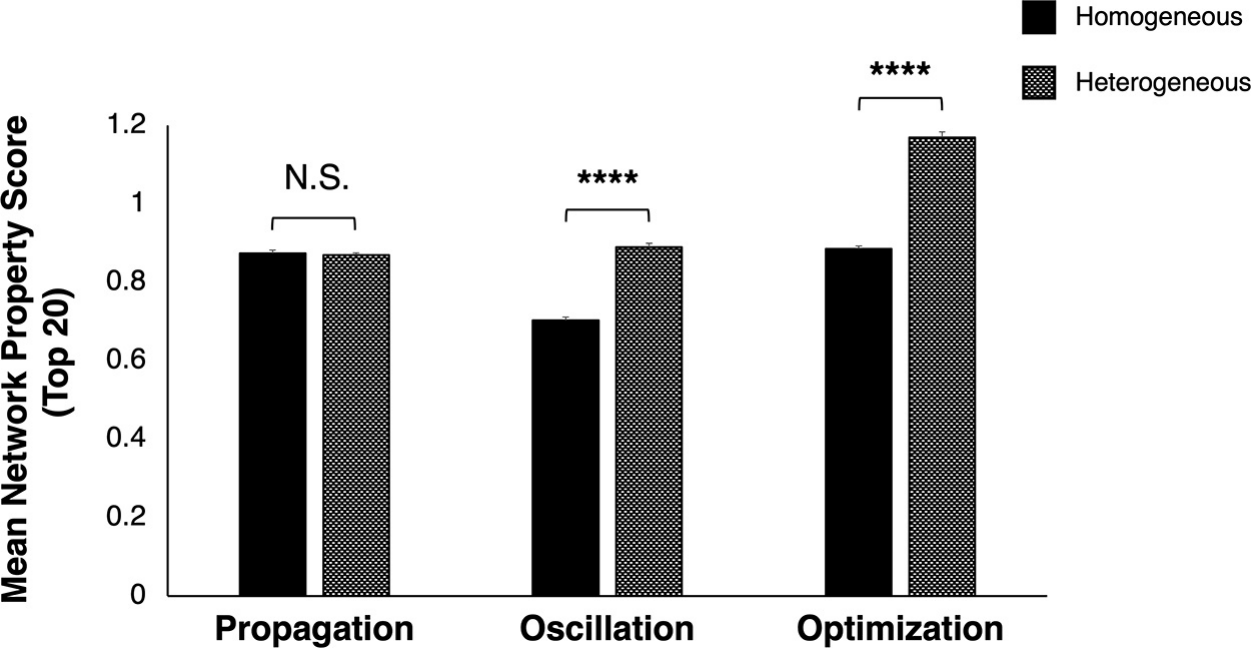
Heterogeneously varied synaptic architectures better supported propagation of oscillation. Propagation, as measured in those network permutations scoring highest with respect to the property, was equally supported in networks where synaptic weights varied independently of one another (heterogeneously; checkered) as in networks where synaptic strength varied homogeneously (black) by class (unpaired *t* test, *t*_(38)_ = 0.46, *p *=* *0.647). By contrast, oscillation and optimization scores were significantly higher in top-performing heterogeneous networks than their homogeneous counterparts (oscillation: *t*_(38)_ = 13.88, *p *<* *0.0001; optimization: *t*_(38)_ = 18.04, *p *<* *0.0001). Each bar corresponds to a mean of the top 20 network propagation, oscillation, or optimization scores within each synaptic architecture group; error bars indicate SEM; ****p <* *0.0001; N.S. = not significant.

We analyzed the relative capacities of homogeneously and heterogeneously varied synaptic networks to support propagation, oscillation, and optimization by comparing the 20 highest scores achieved by homogeneous and heterogeneous network permutations with respect to each performance metric. No significant differences in mean propagation scores between top-performing homogeneous and heterogeneous networks were disclosed (unpaired *t* test, *t*_(38)_ = 0.46, *p *=* *0.647; Fig. 4). We attributed this lack of differences to the fact that network permutations in which the synapses TRN_1_→TC_2_ and TRN_2_→TC_3_ were both maximally weighted would be equally capable of supporting robust signal propagation, regardless of whether these synapses were varied homogeneously or heterogeneously. By contrast, top-scoring heterogeneous network variants better supported both oscillation (*t*_(38)_ = 13.88, *p *<* *0.0001) and optimization (*t*_(38)_ = 18.04, *p *<* *0.0001) than their homogeneous counterparts. Because networks supporting the propagation of oscillatory activity would, by definition, score high with respect to optimization, these results not only confirmed that heterogeneous networks were more likely than homogeneous networks to accommodate this oscillatory mechanism, but furthermore disclosed that propagation of oscillation across the thalamocortical network was associated with higher oscillation scores than postinhibitory-driven oscillation in TC_3_, the predominant form of oscillation observed in homogeneous networks.

## Discussion

The presented analysis suggests that open-loop TC-TRN-TC synaptic motifs (Fig. 1*B*, right) can function as a substrate for signal propagation across the thalamus, and by extension, cortical networks without the need for direct corticocortical, intrareticular or corticothalamic connectivity. Postinhibitory rebound mediated by T-type Ca^2+^ channels served as a mediator of both propagation and oscillation in the simulated networks. TRN-TRN connections, either chemical or electrical (Fig. 1*B*, left and middle), diminished horizontal propagation by disrupting the precise timing relationships required to propagate a signal across the network. Models with heterogeneously varied synapses outperformed those whose synapses varied as a class with respect to the propagation of oscillatory activity, consistent with the emerging literature documenting cellular and synaptic heterogeneity in the TRN (Lee et al., 2007; Halassa et al., 2014; Clemente-Perez et al., 2017). These data suggest that widespread propagating thalamic or thalamocortical activity, under both pathologic and physiological conditions, may be mediated, at least in part, by TC-TRN-TC connections. The model makes strong predictions that can be tested physiologically.

Like most of the thalamic (Destexhe et al., 1993, 1996a; Golomb et al., 1996; Bazhenov et al., 1998; Sohal and Huguenard, 1998) and thalamocortical models (Destexhe et al., 1998; Bazhenov et al., 2002; Rogala et al., 2013) that inspired our model, we used single-compartment, Hodgkin–Huxley neurons. While these model cells contribute to the computational parsimony and practicality of network models, particularly where the analysis of network dynamics is prioritized, they neglect the intrinsic cable properties of real neurons and, relatedly, the spatially disparate nature of synaptic integration and heterogeneous expression of intrinsic and synaptic conductances (Dayan and Abbott, 2005; Herz et al., 2006). Such considerations are particularly relevant here relative to dendritic distributions of T- and H-currents in TC neurons (McCormick and Pape, 1990; Destexhe et al., 1998; Williams and Stuart, 2000; Traub et al., 2005) and TRN neurons (Contreras et al., 1993; Destexhe et al., 1996b; Traub et al., 2005; Crandall et al., 2010). Although multicompartment neuronal models incorporating such details could conceivably alter the network dynamics being studied, they were not necessary to simulate the propagation of oscillatory waves seen physiologically (Bal et al., 1995; Destexhe et al., 1996a; Golomb et al., 1996; Bazhenov et al., 1998; Sohal and Huguenard, 1998).

Additionally, the present model omitted explicit corticothalamic and corticoreticular synapses, both of which have been identified and physiologically characterized to varying degrees (Steriade et al., 1972; White and Hersch, 1982; De Curtis et al., 1989; Contreras et al., 1996; Blumenfeld and McCormick, 2000; Zhang and Jones, 2004; Crandall et al., 2015), although the former were effectively amalgamated with both feedforward sensory and modulatory projections to the thalamus in the form of the Poisson-modulated external input we delivered to individual TC neurons. Both forms of feedback have been implicated in the spread of spindle waves and in the maintenance of their synchronization over large distance scales (on the order of the length of the mammalian forebrain) and are furthermore known to drive spindle wave formation and propagation in vivo by polysynaptically recruiting TC neurons via TRN-mediated postinhibitory rebound (Steriade et al., 1972; Roy et al., 1984; Contreras et al., 1996; Contreras and Steriade, 1996; Suga and Ma, 2003; Sillito et al., 2006; Crandall et al., 2015; Sorokin et al., 2017). It should be noted, however, that short-range propagation of spindle waves, which can be elicited in isolated thalamic slice preparations (Bal et al., 1995; Kim et al., 1995), is preserved following decortication, both in vivo and in silico (Contreras et al., 1996; Contreras and Steriade, 1996; Destexhe et al., 1998).

The signaling dynamics observed in our small-scale, broadly feedforward model would undoubtedly be altered by introducing corticothalamic and corticoreticular feedback, as well as corticocortical synapses: in particular, we can predict, based on other modeling studies that have systematically explored the contributions of such connections within fundamentally closed-loop thalamoreticular frameworks (Destexhe et al., 1998; Rogala et al., 2013), that cortical feedback to the dorsal thalamus and TRN would increase the frequency of postinhibitory rebound in relay cells of the former, while also increasing oscillatory activity through the introduction of new recurrent pathways between the thalamus and cortex. While the enhancement of postinhibitory rebound in the thalamus would a priori suggest an enhancement in the extent of signal propagation mediated through open TC-TRN-TC loops, our results demonstrate that the efficiency of such propagation can be attenuated by decohering activity introduced via intrareticular synapses: as such, whether corticothalamic, corticoreticular, and corticocortical synapses dynamics would ultimately enhance open-loop-mediated propagation or decrement it by interfering with the temporal dynamics underlying this form of intrathalamic signaling would heavily depend on, among other things, the degree to which these additional synapses spatially diverged within the dorsal thalamus and TRN. Notwithstanding elaborations of cortical projections within our network model, our present results suggest that open-loop TC-TRN-TC architectures may mediate a novel form of intrinsic thalamic, and by extension, cortical signal propagation that exists independently of top-down modulation (for example, in localized regions of the thalamus where cortical innervation is sparse) and potentially in parallel to the modes of thalamocortical propagation in which both corticofugal pathways and corticocortical synapses are known to participate. Future efforts within our modeling paradigm stand to incorporate reciprocal cortical projections involving one or multiple cortical layers (Destexhe et al., 1998; Bazhenov et al., 2002; Traub et al., 2005; Izhikevich and Edelman, 2008; Rogala et al., 2013).

### Comparison to related computational models and physiological data

Although the production of spindle waves was not an explicit objective of our study, some of the wave dynamics arising in our networks were nevertheless consistent with those inherent to spindle or spindle-like waves. Despite possessing higher degrees of TC→TRN and TRN→TC synaptic divergence and lacking the exclusively open-loop TC-TRN-TC architecture characterizing a subset of our network variants, other isolated thalamic models allowing for longitudinal wave propagation similarly accommodated this propagation along the lattice of interconnected TC and TRN neurons by way of laterally inhibitory TRN-TC synapses (Destexhe et al., 1996a; Golomb et al., 1996; Bazhenov et al., 1998); at short ranges, this mechanism of signal propagation also prevailed in larger-scale thalamocortical models, while corticothalamic projections acted to propagate activity to more distal sites (Destexhe et al., 1998; Destexhe and Sejnowski, 2003). Comparably, recurrently inhibitory TRN-TC synapses have been documented to play a vital role in the generation of oscillatory behavior in the thalamus (von Krosigk et al., 1993; Destexhe and Sejnowski, 2003). The temporal parameters of propagating and oscillation signals in our model also matched some of those previously reported: the mean signal propagation velocity and oscillation frequency measured across homogeneous networks fell within the ranges of spindle wave propagation velocities and intraspindle spike frequencies reported in both physiological and computational spindle wave studies (Andersen and Andersson, 1968; Steriade and Deschenes, 1984; Kim et al., 1995; Destexhe et al., 1996a; Golomb et al., 1996).

The TC neurons in our model network exhibited both tonic and bursting modes of firing, consistent with extensive physiological characterization (Sherman, 2001). The form of signal propagation generated in our networks via open-loop TC-TRN-TC synaptic pathways, which necessarily depended on TRN-driven postinhibitory rebound in the downstream TC neuron, could, in practice, be elicited regardless of whether the upstream TC neuron fired tonically or in bursts. However, bursting in thalamic neurons receiving open-loop inhibition from the TRN is associated with a greater fidelity of transmission to the cortex relative to tonic firing, as was systematically demonstrated in the computational model that directly inspired our present study (Willis et al., 2015); this finding furthermore holds relative to thalamocortical signaling efficiency more generally (Guido et al., 1995; Reinagel et al., 1999, Swadlow and Gusev, 2001; Krahe and Gabbiani, 2004).

One particularly notable point of departure relative to similar network models was the extent to which thalamoreticular, reticulothalamic, and thalamocortical synapses diverged. Although all three classes of synapses are known to diverge significantly and have been observed to target neuronal somata hundreds of microns from their origins (Jones, 1985; Cox et al., 1996, 1997; Crabtree, 1996; Pinault and Deschênes, 1998; Alonso et al., 2001; Miller et al., 2001; Sherman and Guillery, 2001), the TC-TRN, TRN-TC, and TC-Co synapses in our model were constrained to remain strictly local and minimally divergent (or non-divergent, in the case of TC-TRN and TC-Co synapses). With respect to the first two classes of synapses, this constraint was imposed to probe the impact the disynaptic TC-TRN-TC open-loop motifs characterizing a subset of network permutations, which constituted one of the foci of our study, and analyze the signal propagation they may support. This neuroanatomical scheme contrasted with previous computational models featuring parallel, interconnected thalamoreticular pathways, in which both TC and TRN synapsed bidirectionally with several neighboring TRN and TC cells, respectively, within a radius of several hundred microns (Destexhe et al., 1996a, 1998; Golomb et al., 1996; Bazhenov et al., 1998; Sohal and Huguenard, 1998; Sohal et al., 2000; Traub et al., 2005; Izhikevich and Edelman, 2008). Furthermore, the limited synaptic divergence constrained the spatial and temporal scales over which propagating and oscillating signals persisted in our model. This does not necessarily imply that thalamic and/or cortical signal propagation mediated through open-loop TC-TRN-TC architectures would be inherently limited in either distance or duration, particularly when accounting for the comparative diversity and complexity in the spatial and temporal profiles of real sensory information integrated by the thalamus relative to the highly focal, time-fixed external stimulus approximations we employed to initialize responses reliably across simulations in our model networks, consistent with similarly simplified stimulus representations used in other thalamic or thalamocortical models (Destexhe et al., 1996a; Golomb et al., 1996; Bazhenov et al., 1998; Sohal and Huguenard, 1998; Traub et al., 2005). In light of the limits on the spatiotemporal coherence of signals intrinsic to our present model, however, we would not predict any qualitative changes in propagative or oscillatory dynamics were we to increase the length of our baseline network as presently constituted by adding in parallel additional TC, TRN, and Co neurons.

### The functional implications of open-loop TC-TRN-TC synaptic motifs

The spread of activity from one cortical region to another is a foundational concept at the core of our understanding of sensory processing, higher-order cognitive functions such as attention and language, sleep-related oscillatory phenomena, and pathologic findings such as propagation of ictal discharges and migraine. It has long been speculated that the TRN could serve as a control point for large-scale cortical signal processing given its central location, the high degree of convergence of projections involved in attention, arousal, and emotion onto the TRN, and the TRN’s particularly strong control over TC firing properties (Yingling and Skinner, 1976; Crick, 1984; Guillery et al., 1998; Brunia and Van Boxtel, 2001; McAlonan et al., 2006; John et al., 2013). Here, we showed that open-loop TC-TRN-TC architectures can support at least short-range thalamocortical signal propagation. Within the thalamus, these configurations have thus far been observed both within and across individual thalamic nuclei and are thought to serve as pathways for intra- and cross-modal modulation, respectively (Crabtree et al., 1998; Pinault and Deschênes, 1998; Crabtree and Isaac, 2002; Lam and Sherman, 2005, 2015; Kimura et al., 2007; Lee et al., 2010; Kimura, 2014); as has been previously surmised, these synaptic pathways could also plausibly lend themselves to sensory enhancement, multisensory integration, and attentional mechanisms (Crabtree and Isaac, 2002; Pinault, 2004; Willis et al., 2015; Crabtree, 2018). At a minimum, and as inferred from physiological studies, open-loop pathways should be fully capable of supporting signaling propagation from one thalamic relay neuron to another through a limited number of intervening synapses (with a disynaptic pathway serving as the shortest such configuration).

It should be emphasized that the specific functional roles of open-loop TC-TRN-TC pathways are likely to depend on their densities and distributions within the thalamus (Halassa and Acsády, 2016). If the morphologic, intrinsic, and synaptic heterogeneity of TRN neurons are any indication (Scheibel and Scheibel, 1966; Jones, 1975; Spreafico et al., 1991; Cox et al., 1996; Lee et al., 2007; Halassa et al., 2014; Clemente-Perez et al., 2017), it is reasonable to assume that both TC-TRN and TRN-TC synapses are distributed in a broadly heterogeneous manner across the thalamus. As underscored by our analysis, such synaptic heterogeneity is seemingly a prerequisite for the propagation of oscillatory signals, which, in the case of spindling, can occur in the thalamus independently of cortical involvement (von Krosigk et al., 1993; Bal et al., 1995; Kim et al., 1995) and necessarily involves both recurrently and laterally projecting TRN-TC synapses, the latter of which form of the basis of open loops; outside of this particular functional context, synaptic heterogeneity is broadly speculated to improve the versatility, efficiency, speed, and metabolic economy associated with signal processing (Lengler et al., 2013). If open-loop TC-TRN-TC architectures are indeed to be found within a larger, synaptically diverse thalamoreticular milieu, characterized by variable synaptic divergence and differing densities of sensory, cortical, and other extrinsic innervation, it is moreover reasonable to expect that the degree to which the mode of propagation mediated by these synaptic motifs prevails would vary across thalamic and cortical regions.

To what extent might the functionality of open loops between the dorsal thalamus and TRN depend on arousal state? While sleep and other depressed states of consciousness (e.g., those pharmacologically induced) are associated with thalamic hyperpolarization and therefore tend to amplify both postinhibitory rebound, which underlay the signal propagation in our model, and, by extension, low-threshold bursting (Steriade et al., 1993; McCormick and Bal, 1997; Weyand et al., 2001; Urbain et al., 2019), both phenomena have also been documented in the thalamic relay neurons of awake animals (Guido and Weyand, 1995; Fanselow et al., 2001; Ortuño et al., 2014), and then sometimes selectively in response to particular stimuli (Lesica and Stanley, 2004; Wang et al., 2007). Thus, while there is no mechanistic basis on which to assume that propagation through open TC-TRN-TC loops would be restricted to a particular state of wakefulness, the widespread modulatory afferents received by both the thalamus and TRN from brain areas including the prefrontal cortex, basal forebrain, amygdala, and brainstem leave little doubt that any form of intrathalamic or cortical signaling supported by these synaptic architectures would be highly state-dependent (Halassa et al., 2014). Both forthcoming physiological investigation and future modeling studies will be able to evaluate such predictions and help provide a full accounting of the role of the various modes of connectivity between brain regions.

## Synthesis

Reviewing Editor: Tatyana Sharpee, The Salk Institute for Biological Studies Decisions are customarily a result of the Reviewing Editor and the peer reviewers coming together and discussing their recommendations until a consensus is reached. When revisions are invited, a fact-based synthesis statement explaining their decision and outlining what is needed to prepare a revision will be listed below. The following reviewer(s) agreed to reveal their identity: Yohan John, Laszlo Acsady.

The manuscript shows a clear mechanism by which thalamus and TRN can contribute to propagation of signals in cortex. This has relevance for sleep processing as well as abnormalities in cortico-thalamic signaling that may accompany psychiatric disorders.

Reviewer 1:

The paper presents a new computational model of thalamocortical signaling, implemented using a Hodgkin-Huxley framework. Key parameters were drawn from published electrophysiology data. The goal is to contrast open-loop (also known as lateral inhibition or “off-surround”) and closed-loop (or “off-center”) patterns of connectivity between the thalamic reticular nucleus (TRN) and the dorsal thalamus. The simulations show that (1) open-loop connectivity is most conducive to propagation of thalamocortical signals and generation of synchronous oscillations, (2) TRN-TRN synapses were not crucial to propagation or oscillation, and (3) heterogeneous synaptic networks are more robust than homogeneous networks.

Major comments:

The paper is clearly written and describes results that should be of wide interest. A few additions will improve clarity and help the reader understand the wider implications.

Thalamic activity is often described in terms of phasic and tonic modes. For example, alpha oscillations that depend on low-threshold T-type calcium channels require hyperpolarization, which is associated with certain sleep states. Propagation in the model relies on post-inhibitory rebound - does this require sleep-related hyperpolarization? Some discussion of the phasic-versus-tonic issue and the relation to sleep would improve the Discussion.

A related issue is that propagation of cortico-cortical signals is altered in deep sleep (e.g. Massimimi et al., 2012). It seems as if the model is poised to suggest possible explanations of this phenomenon in the Discussion. For that, elaborating on the absence of cortico-cortical and cortico-thalamic excitation is important, along with comments on how propagation and oscillation might be altered if these excitatory signals were added. Ideally, some simulations of this sort could be added.

Some elaboration on the possible functional importance of heterogeneity might be useful. Is heterogeneity related to some kind of ‘symmetry-breaking’ that makes propagation easier? Some discussion of how heterogeneity boosts propagation and oscillation might be useful.

Minor comments:

The letters ‘A’, ‘B’ and ‘C’ are used for both subplots and neuron labels. This can be a little confusing. Consider using different letters/labels for each.

Line 157: What is the significance of the spindle-like waves in this context? If sleep spindles are being modeled, some comment on the function of propagation might be interesting and helpful.

Figure 1: What do the yellow arrows in subplot D convey? A small addition to the caption would help.

Figure 2: It is not clear what the color coding in subplot A represents. Also, would a set of 3 regression lines convey the same information?

Line 913: Figure 3 caption. The connection between synapses and regression coefficients is a little hard to understand. Do positive and negative values correspond to leftward and rightward propagation? The phrases “property-incrementing” and “property-decrementing” are somewhat obscure, and are not used elsewhere in the text.

References:

Massimini, M., Ferrarelli, F., Sarasso, S., & Tononi, G. (2012). Cortical mechanisms of loss of consciousness: insight from TMS/EEG studies. Archives italiennes de biologie, 150(2/3), 44-55.

Reviewer 2:

In the present in silico paper the effect of intrathalamic architecture is studied on signal propagation and oscillation in the thalamocortical system. Thalamocortical models are used extensively to study the emergent properties of the interacting cell types. The great strength and novelty of the present approach is that using a supercomputing platform the authors systematically examine how changing the properties of the intrathalamic connections influences the behavior of the system. This is critical since concerning at least two major morpho-functional variables (synaptic intra-TRN connectivity, and the “openness” of the TRN-TC circuit) there is no consensus in the literature. The authors establish that open loop TC-TRN circuit support signal propagation but changing intra-TRN connectivity has little effect and state that signal propagation and oscillations are not supported by similar network properties.

1) I could not find the description of some basic properties of the network. They may be present in other works (or in the code) but I would advise to briefly sketch it in the main text. More specifically:

a) How many TC/TRN/L4 neurons do the network have? Does size of the network influence the results?

b) How many TC cells are contacted by a single TRN neuron and how many TRN neurons are connected by a single TC cells? Does “connectedness” effect the results?

c) How “openness” is actually changed? By varying the proportion of TRN cells within the TRN population participating in open or closed loop configuration or by varying the collaterals of individual TRN neurons to participate in open or closed loop configuration?

d) How many gap junctions were there among the TRN cells? What exactly increasing electrical coupling meant? Increasing the strength of contacts or the proportion of contacted TRN cells? Were GJ among TC cells considered?

e) How cortico-thalamic cells are modeled? Do L4 cells project back, or it’s a separate class of cells? Do cortico-thalamic cells innervate both TRN and TC cells?

2) The abstract mention the lack of internal connections in the thalamus. Was it specifically tested what happens if we introduce connections between the TC cells?

3) Cortical inputs. This is where the present work is mostly at odds with the experimental data (line 293-295 and 403-404). These data are not shown and the discrepancy between the experimental and in silico data are not discussed. I propose either to omit these data or fully demonstrate.

4) I would love to see Figure 2 C with Electrical coupling and Openness.

5) It is somewhat unclear to me why there is no systematic change in the oscillation and propagation scores when intra-TRN connectivity (both synaptic and electrical) are changed (see first two columns of Fig 2A).

6) In real life the network parameters explored here are likely variable among different TRN sectors. Can we predict then based on these results that different thalamic regions support oscillation vs propagation to a different degree?

Minor comments:

-Fig 2A is cited only after Fig 2B and 2C.

-For a review on open vs closed TRN loops see Halassa and Acsady 2016 TINS.
